# Personality Traits and Obesity

**DOI:** 10.3390/ijerph16152675

**Published:** 2019-07-26

**Authors:** Jelena Bagnjuk, Hans-Helmut König, André Hajek

**Affiliations:** Department of Health Economics and Health Services Research, University Medical Center Hamburg-Eppendorf, Hamburg 20246, Germany

**Keywords:** obesity, overweight, personality, GSOEP, agreeableness, excess weight

## Abstract

*Objective*: Previous studies investigating the association between body weight and personality traits have found mixed results. This paper uses a large data set and two different study designs (cross-sectional and longitudinal) to provide more consistent estimates of the effect of personality traits on obesity. *Methods*: The present study is based on data from the German Socio-Economic Panel (GSOEP) from the waves 2005 to 2013; GSOEP is a longitudinal survey of private households in Germany that has been carried out since 1984. Responses provided data about personality, measured using the BFI-S Questionnaire, data for self-rated body mass index BMI (to determine the obesity level), as well as information for potential confounders. Cross-sectional and longitudinal logistic regression models were used. *Results*: The cross-sectional study yielded statistically significant results for the association between the outcome variable and four personality factors neuroticism, extraversion, openness and agreeableness. After controlling for several potential confounders, the association between obesity and extraversion, openness as well as agreeableness remained; additionally, the personality factor consciousness reached statistical significance. In the longitudinal study, a statistically significant association was found only for two personality factors, namely extraversion and agreeableness. After adding the control variables, the FE-regression yielded an association only for agreeableness (negative). Gender differences were not significant. ***Discussion***: The findings show that results of a cross-sectional study design differ from the outcome of the longitudinal study design. The latter stresses the association between excess weight and the personality factor agreeableness, contrasting with most outcomes of previous research.

## 1. Introduction

Overweight and obesity have become major public health problems. The WHO estimates the worldwide prevalence of obesity to have nearly doubled between 1980 and 2008. According to country estimates for 2008, over 50% of both men and women in the WHO European Region were overweight [1]. In Australia, nearly two-thirds (63%) of adults were overweight or obese in 2014–2015, with the proportion of those who are obese increasing over time [2]. In the United States the prevalence of obesity in the years 2015–2016 among adults was 39.8% [3]. The number of patients with obesity in hospitals in the populous German state of North Rhine-Westphalia has nearly doubled over a five-year period [4]. This development leads to a dramatic increase in expenses for medical treatment due to secondary health problems of overweight, such as hypertension, diabetes and certain cancers. In addition to direct costs reflected in healthcare, there are indirect costs due to lower work productivity. Moreover, there is a negative relationship between obesity and labour market outcomes, such as wages and employment status, as several studies reveal [5,6]. According to the German Obesity Society (DAG), persons with a body mass index (BMI) of 30 are classified as patients with a need for treatment [4].

There are a variety of factors that lead to obesity. Extensive body weight may be the result of genes, metabolism, behaviour, environment, culture and socioeconomic status [7]. Some observations indicate that rising obesity rates coincide with increases in leisure time and increased fruit and vegetable availability [8], changes in communication patterns and personal contact [9].

Some studies show that body weight is also associated with common personality traits [7,10]. Some physicians consider the behaviour “eating too much” to be the most important risk factor for obesity, ranked above genetic and environmental factors [11], or see their obese patients as lacking self-control [12]. Researchers found statistically significant positive association between addictive eating and BMI [13].

While there is comprehensive research data about the role of genetics, in the last few years, researchers have shown more interest in the association of obesity and personality [14]. However, the understanding of the causes of obesity, individual differences in long-term obesity risk or the likelihood of reversion from obese to non-obese remain unclear [10,15]. Mostly cross-sectional analyses have been conducted [7,16,17] and the results are not consistent, partly because of different personality models and thereby different instruments of investigation used, and partly due to the characteristics of study samples [14]. Faith et al. argue that the quality of instruments for the measurement of data as well as small or clinical samples have to date delivered non-generalizable results [17].

The present study aims to contribute to the topic of obesity by analysing the association between personality traits (according to the Five-Factor-Model (FFM) of personality), and obesity as well as the probability of the shift from non-obese to obese state, using a large and representative longitudinal sample of population in Germany.

There is no common researchers’ opinion with respect to whether an individual’s personality traits are stable or change throughout the life span. McCrae and Costa have stated in the early 90s that individual differences in personality traits are “fixed” by age 30 [18]. However, recent empirical research shows evidence for amenability of personality traits well beyond this age [15,19,20]. Life-span theories do not specify the age at which personality stops developing [21], but it was found that personality traits appear to be relatively stable among working-age adults [22] and the range of changeability declines as people grow older [23]. Moreover, a review of over 80 longitudinal studies revealed that some people are more prone to change than others [21]. Further research found that the trait agreeableness in particular, as well as a facet of conscientiousness, tend to change with age, whereas neuroticism traits tend to reduce across early adulthood and middle age. The smallest changes in adulthood were found in extraversion and openness to experience [24].

## 2. Materials and Methods

### 2.1. Sample

To achieve adequate statistical power, a large sample size with heterogeneous characteristics among the participants is important and allows for the generalization of results to the whole population. Data from the German Socio-Economic Panel (GSOEP) was used to conduct the present study. The GSOEP is a large ongoing annual longitudinal survey of private households and persons in Germany that collects data through personal interviews since 1984, with each wave containing a representative, population-based sample [25]. The survey population includes private households and their members who have reached the age of 17 [26]. The heterogeneous sample comprises individuals with different educational backgrounds, diverse work statuses, different marital statuses, and diverse religious affiliations. The GSOEP aims to collect representative microdata on living conditions, with a focus on sociological questions, but with an increasing emphasis on psychological questions in recent years [19].

The sample used for the present study consists of responses to the 2005 and 2009 waves to the BFI-S personality questionnaires and responses to the 2006 and 2010 waves for the height and weight of panel data participants. The response rate in GSOEP is considered to be very high, e.g.**,** in 2005, the response rate was 94.6% [27], and it was 93.4% in 2010 [28]. Attrition, which appears in almost all questionnaire-based studies and leads to exclusion of respondents due to missing or incomplete information, was low (3.2%), thus it would not bias the results [29].

The cross-sectional analysis in the present study is based on 18,291 observations, and the longitudinal analysis is based on 2026 observations. Please see the statistical analysis section for further details. Ethical approval was not obtained because the criteria for the need for an ethical statement were not met (risk for the respondents, lack of information about the aims of the study, examination of patients). However, the German Council of Science and Humanities (Wissenschaftsrat) evaluated the German Socio-Economic Panel (GSOEP) at the Deutsches Institut für Wirtschaftsforschung, (DIW), Berlin. The German Council of Science and Humanities approved the GSOEP.

### 2.2. Dependent Variables

Data related to the outcome measure BMI was collected every second year from 2002; height and weight were self-assessed and reported by panel survey participants. The BMI (kg/m^2^) was computed from these statements, and individuals were categorized, according to the WHO, as either non-obese or obese (0—non-obese, BMI < 30 kg/m^2^; 1—BMI ≥ 30 kg/m^2^) [30].

### 2.3. Independent Variables

Personality traits according to the Big Five model represent the main independent variables in the present work. The “Big Five” personality traits were assessed using an especially developed short version of common inventories, the BFI-S [29]. Each item consists of one statement. Participants are asked to indicate their agreement on a scale ranging from 1 = “does not apply at all” to 7 = “applies perfectly” [19]. The BFI-S Questionnaire was included in the GSOEP waves of 2005 and 2009. In 2005, nearly 20,000 participants completed the GSOEP Big Five Inventory; in 2009, more than 20,000 participants delivered their responses [31].

Dehne and Schupp tested the reliability and external validation of the BFI-S, and concluded that BFI-S could be regarded as a valid measurement tool for the “Big Five”. In their work, they provide empirical evidence for the validity of the concept [29]. Kanning found a certain potential for error in the evaluation procedure which requires special attention by the evaluation staff. However, the performance objectivity and the evaluation objectivity can be considered as a given [32]. Evidence for measurement equivalence and 5-year retest stability of BFI-S was furthermore demonstrated in the analysis performed by Lang et al. in 2011.

Additionally, in order to test whether some sociodemographic factors affect the association between the personality traits and obesity, the following potential confounders were included in the analysis: age (in years: min 17, max 96), unemployment status (0 = yes, 1 = no), marital status (0 = married and living separated, single, divorced, widowed; 1 = married and living together), self-rated health (from 1 = “very good” to 5 = “bad”), presence of severe disability (0 = no, 1 = yes). Self-reported questionnaires provided data on study control variables. The controlling variables were chosen based on findings of previous research showing an association with obesity or risk to become obese, as well as on the availability of data in the sample (for further details, see [33,34,35,36,37]).

### 2.4. Statistical Analysis

This paper applies a logistic regression model to test for single time point association between FFM personality traits and obesity, following by a conditional Fixed-Effects (FE) logistic regression model to analyse the longitudinal relationship between the personality traits and the probability of a shift from a non-obese state to an obese state. Thus, only individuals were included in FE models when they changed their obesity status in the observation period. Consequently, please note that only 2026 observations were used in FE regression analysis because exclusively these individuals changed their obesity status in the observation period.

Previous studies on this topic are mostly cross-sectional and are based on a defined group of survey participants, whereas the present study uses panel data, allowing for the construction of a longitudinal design. It is difficult to draw causal conclusions from cross-sectional studies, as they have no time dimension [11]. Therefore, longitudinal regression models were used. The Hausman test suggested that FE regressions are the preferred method of choice (Hausman test statistic = 194.66, *p* < 0.001).

Two different models with two variations were developed:

- a cross-sectional model to analyse which personality traits could be associated with obesity in the year 2005, followed by a model adjusting for potential confounders; and
- a longitudinal model to analyse the longitudinal association between personality factors and obesity over the period between 2005 and 2009, followed by a model adjusting for potential confounders and stratified for gender-specific differences

The level of significance was set at 0.05. Analyses were carried out using Stata Version 14.2, Stata Corp., College Station, TX, USA.

## 3. Results

### 3.1. Cross-Sectional Analysis

To examine the cross-sectional associations between personality and obesity, the data from the 2005 and 2006 waves of the GSOEP, the largest household study in Germany, was analysed by means of logistic regression.

Sample characteristics of the individuals included in the logistic regression are shown in Table 1.

The final study sample contained 18,291 observations (due to missing data on some variables, the sample size ranged from 18,398 to 18,291), mean age was 47.37 (±17.28) with the range 17 to 96 years. Obesity prevalence was 16.08%. The average scores for personality factors in the full sample yielded for neuroticism 11.865 (±3.655), for extraversion 14.488 (±3.393), for openness to experience 13.520 (±3.629), for agreeableness 16.380 (±2.926), and for conscientiousness 17.718 (±2.805).

The outcomes of the cross-sectional logistic regression analysis with and without adjustment for confounders are presented in Table 2.

The first model shows positive associations between obesity and the personality traits neuroticism (OR: 1.02) and extraversion (OR: 1.01), and negative associations between obesity and openness to experience (OR: 0.97) and agreeableness (OR: 0.98). After adjusting for controlling variables, the results slightly changed. The positive association with the trait extraversion (OR: 1.03) and negative associations with the traits openness to experience (OR: 0.98) and agreeableness (OR: 0.97) remained. However, the result of the trait conscientiousness (OR: 0.98) became significant, yielding a negative association. Neuroticism did not reach statistical significance.

### 3.2. Longitudinal Analysis

To examine whether personality is associated with weight fluctuations over time (weight measured by means of BMI), a conditional fixed-effects logistic regression, with responses from participants having valid assessments of personality from the waves 2005/2006 and 2009/2010, was conducted to estimate the total standard deviation within each person.

Sample characteristics of the individuals included in the FE regression are shown in Table 3. The final study sample contained 2026 observations (due to missing data on some variables, the sample size ranged from 2026 to 2012). The mean age was 57.78 (±15.84) with the range 17 to 94 years.

The average scores for personality factors in the full sample were 11.706 (±3.626) for neuroticism, 14.415 (±3.359) for extraversion, 13.018 (±3.602) for openness to experience, 16.039 (±2.95) for agreeableness, and 17.48 (±2.816) for conscientiousness.

The outcomes of the FE logistic regression analysis with and without adjustment for confounders are presented in Table 4.

The first model shows that the probability of becoming obese (BMI > 30) decreased with increasing extraversion (OR: 0.95) and increasing agreeableness (OR: 0.94). After adjusting for possible confounders, the outcome variable remained negatively associated only with the trait agreeableness (OR: 0.95). Other traits did not reach statistical significance.

### 3.3. Gender-Specific Interaction

As previous studies found gender-related differences in personality traits [16,24,38,39], it is important to analyse gender-specific variations in the results of the present study. Use of FE regression implies a limitation on time-constant variables. Therefore, to identify whether there is a difference between the effect of the personality trait (PT) agreeableness in men or women, a stratification was conducted. Significance of results can be analysed by means of interaction term included in the FE regression. The results of the analysis for men and women are presented in Table 5. No significant effect of any of the PTs on the risk to become obese for men was found, but a negative effect of neuroticism (OR: 0.94) for women was identified. For both genders, the controlling variable age (male: OR: 1.12; female: OR: 1.16) reached statistical significance, showing a positive association. Moreover, for women, the confounder self-rated health (OR: 1.24) yielded a statistically significant result, showing a positive effect of poor self-rated health on the risk of becoming obese. It is important to note, that for the model stratified for men, no convergence was achieved.

As the analysis conducted separately for men and women yielded different results for the association between the PT agreeableness and the outcome measure, it is important to assess whether the effect of agreeableness on the risk of becoming obese among men differs significantly from the effect of this PT on the risk of becoming obese among women. The calculation of the interaction effect did not deliver significant results, which means that gender does not have a moderating effect in the present study.

## 4. Discussion

Based on GSOEP panel data, the goal of the current study was to investigate a single time-point association between personality traits (according to the FFM) and obesity, followed by a longitudinal association analysis between personality traits and the probability of a shift from a non-obese to an obese state. The data used was taken from the survey waves 2005/2006 and 2009/2010. The data was analysed by means of cross-sectional logistic regression and a conditional FE logistic regression.

Considering the fact that the cross-sectional data is observational only, and that the majority of studies on this topic are based on a cross-sectional design, the purpose of the present analysis was also to determine—using the same data sources—a difference in the results of a cross-sectional and longitudinal constructs. The investigation revealed a big discrepancy in results. A single time-point analysis yielded a statistically significant association between obesity and the personality traits neuroticism, extraversion, openness to experience and agreeableness (prior to adjustment for confounders), as well as with extraversion, openness to experience, agreeableness and conscientiousness (after adjustment). However, the longitudinal study delivered only a statistically significant association between risk of obesity and extraversion and agreeableness (before adjustment for confounders), as well as with agreeableness (after adjustment) respectively. Simply, the association with extraversion was positive in a cross-sectional setting and negative in the longitudinal setting.

The controlling variables age, unemployment status, marital status and self-rated health reached the statistical significance level in the cross-sectional study, whereas the longitudinal study yielded a significance level only for the variable age. This association can be explained by the finding that agreeableness increases with age [24].

Longitudinal analyses help to examine whether, and which, personality traits are predictors of BMI change. The main advantage of using panel data is reducing the problem of unobserved heterogeneity [40]. Taken together, these findings suggest that the probability of shift from non-obese to obese state is negatively influenced by the PT agreeableness. This outcome can be explained by the finding that higher levels of agreeableness may be of benefit to mental health, and could therefore be related to healthier behavioural factors [16,41].

An analysis of an interaction effect for gender did not yield a statistical significance, which shows that the effect of agreeableness on risk to become obese for one gender group (women) is not significantly different from the effect for another gender group (men). Gender had no moderating effect in the present study.

Previous studies analysing an association between body weight measures and personality traits have not demonstrated a clear outcome. The inconsistent findings might be due to the differences in personality measures, samples, or study designs used.

Conscientiousness is one of the traits mostly associated with body weight: conscientious individuals have lower risk for obesity (see e.g., [7,16,42,43,44,45,46]), demonstrate preventative health-related behaviours [15], and are more likely to exercise [47]. Conscientiousness is also considered to be predictive for the reversion of obesity to non-obesity in obese individuals [10]. Surprisingly, one cross-sectional study with overweight and obese women showed conscientiousness to be positively related to BMI. At the same time, conscientiousness was also positively related to cognitive dietary restraint in this sample [16].

Abnormal weight has also been associated with the trait Neuroticism. Some researchers suggest that neuroticism can be associated with both under- and overweight [7,45]. A meta-analysis by Jokela et al. found no evidence for an association between neuroticism and obesity risk. However, subgroup analyses suggested that obesity might be associated with higher neuroticism in European countries and in Australia, but not in the United States [10]. A cross-sectional study found an association between neuroticism and greater adiposity among women, but not men [42]. In the same analysis, extraversion was positively associated with BMI, some associations emerged for agreeableness, and none for openness. Participants who were overweight or obese scored slightly higher on antagonism (low agreeableness) than normal weight participants [42].

Similar to the findings of the present study, the outcomes differ depending on study design. In a large study by Sutin et al., participants with higher neuroticism or extraversion scores or lower conscientiousness scores had higher BMI in a cross-sectional setting. Longitudinally, low agreeableness and impulsivity-related traits predicted greater increase in BMI across the adult life span [7]. The study by Armon et al. also showed that conscientiousness was negatively related to body weight measures in a single time-point analysis, but was no longer associated with changes in this trait over time [45]. This is confirmed by results of the present study. However, in a large meta-analysis by Jokela et al. that included cross-sectional and longitudinal studies, the findings were similar for both designs, indicating that high conscientiousness was associated with a lower risk of developing obesity, and a lower persistence of obesity over time [10].

Some studies found, through stratification of data, gender differences in the association between PTs and overweight [16,45]. However, the difference in estimated values did not reach a statistically significant level.

Several reasons might explain why the results of the present study differ from those in previously conducted investigations. The GSOEP has a large sample size and the characteristics of the population are heterogeneous, whereas most studies are based on defined groups of participants who meet certain criteria. Furthermore, the questionnaire used to determine PTs might influence the results, depending on the number of items per scale or the language used (for details, see [29]). As the overview of previous research indicates, using a different study design and regression type delivers diverse results. The present study confirms this observation: the results of cross-sectional regression indicate associations between BMI and several PTs, while longitudinal regression results show a correlation with only one trait, namely agreeableness.

Of the studies evaluated, the meta-analysis by Jokela et al. has the most similarities with the present work. That is, a large sample size (incl. GSOEP), as well as cross-sectional and longitudinal analysis and the use of FFM of personality traits. In contrast to the present study, the authors used a random effect meta-analysis to estimate the values, and received the same association in the cross-sectional as in the longitudinal setting. Some researchers refer to differences in results due to unobserved heterogeneity [48]. However, panel data, in combination with FE regression, can solve the problem of person-specific unobserved heterogeneity [40]. The results of the current study show the importance of testing health-related associations with PTs using a longitudinal design. This study has measured associations between personality traits and obesity at one time point and over time. While most studies working on this topic are cross-sectional, the present work used a longitudinal approach.

The data used was derived from the population-based longitudinal study of households in Germany (GSOEP), representing a heterogeneous sample. A limitation with regard to the size of the sample needs to be mentioned. Despite the large household number participating in GSOEP, the final sample available for the present study was reduced to 2206 observations, which might affect the degree to which the survey is representative. When using a fixed effects regression, it is important to deal with one constraint, namely, that it cannot estimate time-invariant variables, e.g., gender. Therefore, an additional separate analysis for men and women was conducted. Thanks to the longitudinal nature of panel data, and the use of the FE regression approach, it was possible to reduce unobserved intrapersonal heterogeneity [40]. However, the longitudinal design was based on only two periods of measurement. Considering the possibility of curvilinear terms, more data measurements are needed [45]. Currently, no further assessments of the PTs in the GSOEP are available.

Self-reported trait scales include random and systematic errors; however, since the purpose in the present work was not to measure or compare levels in an absolute sense, this bias can be considered irrelevant [49]. Moreover, spouse ratings on the Big Five correlate with self-ratings to a high extent [50]. This research focussed on BMI as an outcome measure, using self-reported weight and height. Respondents tend to over- or underestimate their own measures [51]; however, there is a high correlation between reported and measured weight and height [52]. Moreover, at several measurements over time, respondents tend to misreport data in the same direction [42]; therefore, it would not bias the FE estimations.

The FFM model has been criticized due to the broadness of each dimension [14,53]. Some researchers propose a model with six [54] or even seven PTs [55] to better specify each trait. However, a large body of research has demonstrated the reliability of the Big Five, and it is one of the most used concepts in personality research [44], enabling comparison with other studies. As for the BFI-S questionnaire, it should be noted that its Cronbach’s alpha value fell below the commonly used cut-off value of 0.7, due to the small number of items. Nevertheless, after testing for reliability and validity, it is considered a valid tool for surveys [29]. Indeed, it has been said that “to be reliable, ratings must be simple” [50].

With respect to the control variables used, due to the limited scope of this work and the incompleteness of the data available, not all possible confounders could be included in the study. The tendency to overweight is strongly related to family home. Comparisons of parents with higher and lower levels of education showed that significantly more children of parents with lower levels of education are overweight or obese [56]. A study on mental disorders showed that adults with depression have a higher chance of suffering from obesity [57]. A review on alcohol consumption also revealed that higher intakes of alcohol in the absence of alcohol dependence may increase the risk of obesity [58]. Therefore, future research should consider chronic diseases, mental health, alcohol consumption, and education level as additional variables. Reverse causality (e.g., whether obesity status has predicted a change in personality [10,42]) should also be considered in future studies.

## 5. Conclusions

Obese individuals face multiple forms of prejudice because of their weight and perceive disparities in important areas of living, including employment, health care, and education [59]. This paper set out to examine the association between personality traits and obesity in cross-sectional and longitudinal settings, based on a nationally representative sample of the German population (GSOEP). Obesity was found to be associated with four personality traits in a cross-sectional study; however, this effect was no longer observed when using the same data in a longitudinal setting. The only trait found to be associated (negatively) with the risk to become obese was agreeableness, adjusted for potential confounders.

Given the development of obesity is one of the major public health issues today, the results of the present study might serve a basis for relevant decisions in this area.

## Figures and Tables

**Table 1:** Sample characteristics for individuals included in logistic regression (*n* = 18,291; year 2005)

Variable	N/Mean	%/(SD)
Age in years (min-max)	47.36 (17–96)	(17.28)
Marital status		
Married	7287	39.84%
living separated, single, divorced, widowed Married and living together	11,004	60.16%
Unemployment status		
Unemployed	1314	7.18%
Employed	16,977	92.82%
Self-rated health		
(1 = very good–5 = bad)	2.60	(0.95)
Severe disability		
No	16,151	88.3%
Yes	2140	11.7%
Obesity prevalence		
No	15,350	83.92%
Yes	2941	16.08%
FFM Personality traits		
Neuroticism (min-max)	11.865 (3–21)	(3.655)
Extraversion (min-max)	14.488 (3–21)	(3.393)
Openness to experience (min-max)	13.520 (3–21)	(3.629)
Agreeableness (min-max)	16.380 (3–21)	(2.926)
Conscientiousness (min-max)	17.718 (3–21)	(2.805)

**Table 2:** Results of cross-sectional logistic regression analysis of the association between personality traits and obesity (0 = non-obese, 1 = obese); data from wave 2005/2006.

Independent Variables	Outcome Measure Pure Results, OR (95% Confidence Interval)	Outcome Measure Results with Confounders, OR (95% Confidence Interval)
Neuroticism	1.02 *** (1.01–1.03)	0.99 ^+^ (0.97–1.00)
Extraversion	1.01 * (1.00–1.03)	1.03 *** (1.02–1.04)
Openness to experience	0.97 *** (0.95–0.98)	0.98 *** (0.97–0.99)
Agreeableness	0.98 *** (0.96–0.99)	0.97 *** (0.96–0.99)
Conscientiousness	0.99 (0.98–1.01)	0.98 ** (0.96–0.99)
Age		1.01 *** (1.01–1.01)
Marital status (Ref.: Married and living separated, single, divorced, widowed)		1.42 *** (1.30–1.56)
Unemployment status (Ref.: Unemployed)		0.68 *** (0.58–0.78)
Self-rated health (Ref.: from 1 = “very good” to 5 = “bad”)		1.40 *** (1.33–1.47)
Severe disability (Ref.: No)		1.12 ^+^ (0.99–1.26)
Number of observations	18,398	18,291
Pseudo R^2^	0.0046	0.0414

Comments: * *p* < 0.05, ** *p* < 0.01, *** *p* < 0.001, ^+^ *p* < 0.10.

**Table 3:** Sample characteristics for individuals included in conditional fixed effect logistic regression (*n* = 2026; year 2005, 2009).

Variables	N/Mean	%/(SD)
Age in years (min-max)	57.78 (17–94)	(15.84)
Marital status		
Married and living separated, single, divorced, widowed	680	33.56%
Married and living together	1346	66.44%
Unemployment status		
Unemployed	154	7.6%
Employed	1872	92.4%
Self-rated health		
(1 = very good–5 = bad)	2.83	(0.94)
Severe disability		
No	1718	85.09%
Yes	301	14.91%
Obesity prevalence		
No	1013	50%
Yes	1013	50%
FFM Personality traits		
Neuroticism (min-max)	11.706 (3–21)	(3.626)
Extraversion (min-max)	14.415 (3–21)	(3.359)
Openness to experience (min-max)	13.018 (3–21)	(3.602)
Agreeableness (min-max)	16.039 (3–21)	(2.95)
Conscientiousness (min-max)	17.48 (6–21)	(2.816)

**Table 4:** Results of conditional fixed effects logistic regression models (data from waves 2005/2006 and 2009/2010), with outcome measure “Obesity next = shift from non-obese to obese state”.

Independent Variables	Outcome Measure Pure Results, OR (95% Confidence Interval)	Outcome Measure Results with Confounders, OR (95% Confidence Interval)
Neuroticism	0.97 (0.94–1.01)	0.99 (0.95–1.02)
Extraversion	0.95 * (0.91–0.99)	0.97 (0.93–1.01)
Openness to experience	1.06 (0.98–1.05)	1.03 (0.99–1.08)
Agreeableness	0.94 ** (0.90–0.98)	0.95 * (0.91–0.99)
Conscientiousness	0.99 (0.95–1.04)	1.00 (0.95–1.05)
Age		1.14 *** (1.10–1.18)
Marital status (Ref.: Married and living separated, single, divorced, widowed)		1.28 (0.82–1.98)
Unemployment status (Ref.: Unemployed)		0.86 (0.54–1.37)
Self-rated health (Ref.: from 1 = “very good” to 5 = “bad”)		1.05 (0.91–1.22)
Severe disability (Ref.: No)		0.60 + (0.34–1.06)
Number of observations	2026	2012
Pseudo R^2^	0.114	0.0574

Comments: * *p* <0.05, ** *p* < 0.01, *** *p* < 0.001, ^+^ *p* < 0.10.

**Table 5:** Results of gender stratification (wave 2005/2006 and 2009/2010), with outcome measure “Obesity next = shift from non-obese to obese state”.

Independent Variables	Outcome Measure Male OR (95% Confidence Interval)	Outcome Measure Female OR (95% Confidence Interval)
Neuroticism	1.02 (0.97–1.08)	0.94 * (0.89–1.00)
Extraversion	0.94 ^+^ (0.89–1.00)	1.00 (0.94–1.08)
Openness to experience	1.04 (0.98–1.10)	1.02 (0.96–1.08)
Agreeableness	0.94 ^+^ (0.89–1.00)	0.97 (0.90–1.03)
Conscientiousness	1.00 (0.94–1.07)	0.99 (0.92–1.06)
Age	1.12 *** (1.07–1.17)	1.16 ***(1.10–1.22)
Marital status (Ref.: Married and living separated, single, divorced, widowed)	1.54 (0.83–2.84)	0.96 (0.50–1.86)
Unemployment status (Ref.: Unemployed)	0.82 (0.45–1.50)	0.89 (0.41–1.92)
Self-rated health (Ref.: from 1 = “very good” to 5 = “bad”)	0.90 (0.73–1.10)	1.24 * (1.00–1.52)
Severe disability (Ref.: No)	0.71 (0.34–1.47)	0.49 (0.191.24)
Number of observations	1062	950

Comments: * *p* < 0.05, ** *p* < 0.01, *** *p* < 0.001, ^+^ *p* < 0.10.
